# Microstructure and Mechanical Properties of Zinc Matrix Biodegradable Composites Reinforced by Graphene

**DOI:** 10.3389/fbioe.2021.635338

**Published:** 2021-03-31

**Authors:** Qianfei Dai, Shanshan Peng, Zongkui Zhang, Yuan Liu, Mei Fan, Fei Zhao

**Affiliations:** ^1^College of Materials and Metallurgy, Guizhou University, Guiyang, China; ^2^Key Laboratory for Materials Structure and Strength of Guizhou Province, Guiyang, China; ^3^Hospital of Guizhou University, Guiyang, China

**Keywords:** zinc matrix composites, GNS, mechanical properties, microstructure, biodegradable

## Abstract

This work used spark plasma sintering (SPS) to prepare graphene nanosheets (GNS) reinforced zinc matrix composites. The influence of GNS on the microstructure and mechanical properties of zinc matrix composites was studied. The results show that the GNS/Zn composites prepared by SPS have a dense structure and good interface bonding, and GNS are uniformly distributed in the zinc matrix. Adding GNS can significantly improve the mechanical properties of the zinc matrix. When 0.7 wt% GNS are added, the comprehensive mechanical properties of the composite material are improved. The ultimate tensile strength is 254 MPa, and the Vickers hardness is 65 HV, which are 126 and 20.3% higher than those of pure zinc (112 MPa and 54 HV), respectively. The strengthening mechanisms of GNS/Zn composites are mainly load transfer of GNS and dislocation strengthening caused by coefficient of thermal expansion (CTE) mismatch. In addition, the biodegradability of GNS/Zn composites was evaluated by electrochemical measurement and immersion test. The results show that adding GNS to the zinc matrix will accelerate the degradation rate of the composite material. But the degradation rate can be controlled by the content of GNS. Its degradation rate is in the range of 69–301 μm/a, an ideal degradation rate as an orthopedic implant material.

## Introduction

Metal-based biomedical materials are widely used for clinical applications because of their good mechanical properties and processing properties. At present, metal implant devices mainly include titanium alloy, stainless steel and cobalt-based alloy (Yuan et al., 2019). The above mentioned implanted devices have good corrosion resistance and can maintain the stability of the structure and performance in the body for a long time. When the device is completed in service, it needs to be removed by a second operation, which increases the medical cost and causes secondary harm to the human body. However, as a short-term implant material, the implanted device needs to be able to corrode and degrade during treatment while still maintaining specific functions, and the material itself and the degradation products are required to be absorbed by the body or excreted by metabolism (Shih et al., 2010; Li et al., 2018). The implanted devices cannot only meet the treatment requirements but also avoid the pain and cost increase caused by the second operation. Biodegradable zinc-based materials are candidates for a new generation of orthopedic implants. Compared with magnesium, biodegradable zinc does not produce hydrogen cavitation corrosion caused by rapid corrosion (Wei et al., 2020). Compared with nondegradable metals such as iron and titanium, zinc exhibits a better degradation rate in the body (Arjmand et al., 2019). In addition, zinc can promote the growth of bone tissue and play an important role in the process of bone mineralization and bone formation, and zinc also participates in a large number of physiological reactions of the human body, including cell development, gene expression, the immune system, and the nervous system (Gao et al., 2020; Qu et al., 2020; Racca et al., 2020).

However, the room temperature mechanical properties of pure zinc are poor, and the mechanical properties of zinc cannot meet the requirements of the mechanical properties of implanted medical biological bone internal fixation materials with a tensile strength higher than 200 MPa and elongation higher than 10%. Alloying is currently one of the main ways to improve the mechanical properties of zinc alloys. Vojtěch et al. (2011) prepared Zn/1 Mg alloy by adding alloying element Mg to the Zn matrix. Its strength increased from 90 MPa to 190 MPa, but the fracture elongation decreased to 2%. Niu et al. (2016), added 1.0 wt% Mg to Zn/3.0 Cu alloy, and the yield strength and ultimate tensile strength increased from 214 and 250 MPa to 427 and 440 MPa, respectively, while the elongation dropped sharply from 47 to 1%. Although the alloying method enhances the strength of zinc, the plasticity will be greatly reduced, which seriously affects the application of the material. At present, the preparation of metal matrix composites (MMCs) is an effective way to improve the comprehensive mechanical properties of metals. The mechanical properties can be effectively improved by selecting an appropriate reinforcement and preparation method.

Graphene is a two-dimensional carbon nanomaterial with a hexagonal honeycomb structure composed of C atoms and sp2 hybrid orbitals. It has excellent mechanical properties and special thermal, optical and electrical properties (Zhang et al., 2016; Guirguis et al., 2020; Omidian et al., 2020). It is considered a revolutionary material in the field of materials (Papageorgiou et al., 2017). Therefore, how to use graphene as reinforcement to achieve a good combination of material strength and plasticity has become a hot topic for researchers. Among them, the preparation of GNS-reinforced MMCs by powder metallurgy is an important research direction. Chen et al. (2019) prepared GNS-reinforced magnesium-based composites by thixotropic molding. When the GNS content is 0.6 wt%, the mechanical properties are the best, with the hardness and tensile strength reaching 92.3 HV and 245 MPa, respectively. Rashad et al. (2015) showed that the effect of graphene prepared by the semipowder method on the mechanical properties of pure aluminum increased the yield strength and tensile strength of 0.3 wt% GNS composites by 14.7, 11.1, and 11.8% compared with pure aluminum. Bhadauria et al. (2019) used SPS to prepare graphene-reinforced nano-aluminum-based composite materials, adding 0.5 wt% GNS to the nanocrystalline Al matrix, and its yield strength and ultimate tensile strength increased by 85 and 44%, respectively. For the same content of GNS microcrystalline aluminum, the elongation when the matrix composites break was as high as 29%. It can be seen that graphene as a reinforcement method can effectively improve the comprehensive mechanical properties of composite materials, pointing out a new direction for the improvement of the mechanical properties of zinc. However, graphene-reinforced MMCs still have problems such as uneven graphene dispersion and weak interface bonding.

In this study, the surface of GNS was modified by surfactants, and GNS were dispersed in combination with ball milling. Spark plasma sintering (SPS) is used to prepare graphene nanosheet-reinforced zinc-based composite materials. The study focused on the influence of graphene nanosheets (GNS) on the structure and properties of zinc-based composites and clarified the mechanisms of graphene nanosheet strengthening of zinc-based composites.

## Materials and Methods

### Raw Materials

GNS are a gray black powder with a specific surface area of 50–200 m2/g, a thickness of 1–3 layers (0.686–1.054 nm), a single layer rate >80%, and a diameter to thickness ratio of 9,500, from Shenzhen Zhongsen Linghang Technology Co., Ltd. The zinc powder has a purity of 99.5% and an average grain size of 5μm from Zhongnuo New Material Technology Co., Ltd., Beijing.

### Preparation of GNS/Zn Composite Materials

**FIGURE 1:**
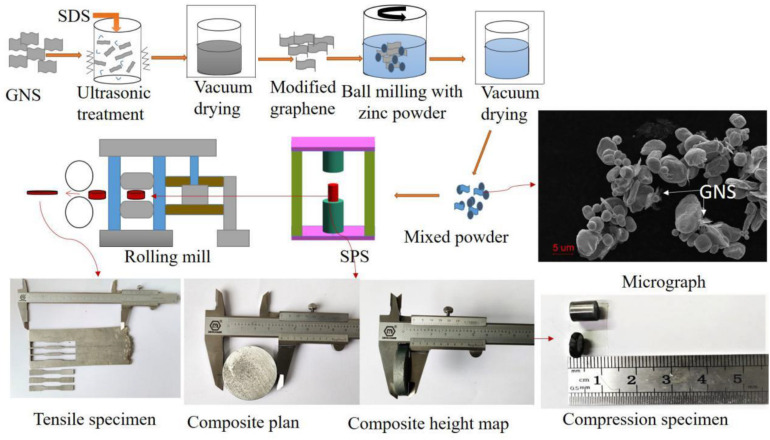
Preparation of GNS/Zn composite materials.

Figure 1 is a schematic diagram of the preparation process of GNS/Zn composites. GNS were added to deionized water for ultrasonic cleaning for 2 h, and sodium dodecyl sulfate (SDS) was gradually added dropwise during the cleaning process to modify the surface of GNS. Then, absolute ethanol was used as a control agent, zinc powder was added to a 300 r/min ball mill (QM-3SP2) for 6 h, and vacuum drying was performed for 8 h to obtain GNS/Zn powder. The GNS/Zn powder was placed in a graphite mold with an inner diameter of Φ60 mm, and the powder was fixed with graphite paper and densified by spark plasma sintering (SPS-625HF). Pure zinc was also sintered using the same process as a reference. The plasma sintering process included the following parameters: sintering temperature of 340°C, heating rate of 100°C/min, sintering pressure of 45 MPa, holding time of 10 min, and a sample size of Φ60 mm × 7 mm for the cylindrical GNS/Zn composite material.

### Rolling Process

The crystal structure of zinc is a close-packed hexagonal structure, which is brittle. To further improve the interface bonding strength of the composite material, 6061 aluminum alloy was used to wrap and roll the composite material. All the samples were processed by using a wire cutter to cut them into plates with a size of 50 mm × 40 mm × 7 mm for multi-pass hot deformation rolling, and the remaining materials were used for organization and structure analysis. The schematic diagram and actual size of the sample are shown in Figure 1. The rolling process is shown in Table 1.

**TABLE 1:** Rolling process of composite materials.

Temperature/°C	Stage one	Stage two	Stage three	Total deformation
380	Four passes, single pass rolling deformation 5%	Four passes, single pass rolling deformation 10%	Four passes, single pass rolling deformation 5%	80%

### Characterization of the Structure and Performance of the Materials

Using a Fourier transform infrared spectrometer (FTIR, Nicolet 670), samples of both pristine and physio-chemically fictionalized GNS, in succession was mixed with FTIR grade KBr and pressed in the form of pellets. Measurements were taken in transmission mode in a wave number range of 4,000–500 cm^–1^. X-ray diffractometer (XRD, X, Pert PRO MPD) using CuK a radiation was adopted to identify the constituent phases of pure Zn and GNS/Zn composites with scanning range from 15°to 90°and scan rate of 15°min^–1^. Laser microscopic confocal Raman spectroscopy (Raman, Renishaw lnvia), the test range wave number is 1,200–3,000 and the laser wavelength is 325, the phase composition of the composite material and the integrity of GNS is analyzed. Metal ionic bonds in composites were detected using XPS (Avantage) and the experimental standard was Mono AlKa energy of 1,486.6 eV. Scanning electron microscopy (SEM.JEOL JSM-7500F), energy spectroscopy (Se3400N) and transmission electron microscopy (TEM, JEOL2100) characterize the microstructure and structure of the composite material. The microhardness of the samples was tested with a microhardness tester (HV-1000), 0.1 KN for 10 s, and each sample was repeated 5 times. Set up three parallel samples. The tensile and compressive tests at room temperature were carried out on Tse-104B universal testing machine. Total length of tensile dimension is 65 mm, the length of parallel segment is 15 mm, the length of the arc segment is 18.3 mm, and the tensile rate is 0.5 mm min^–1^. The diameter of standard compression specimen is 5 mm, the length is 8 mm, and the compression rate is 2 × 10^–4^/s. There are at least three parallel specimens in each group.

## Results and Discussion

### Morphology and Microstructure of the Powder and GNS/Zn Composite

**FIGURE 2:**
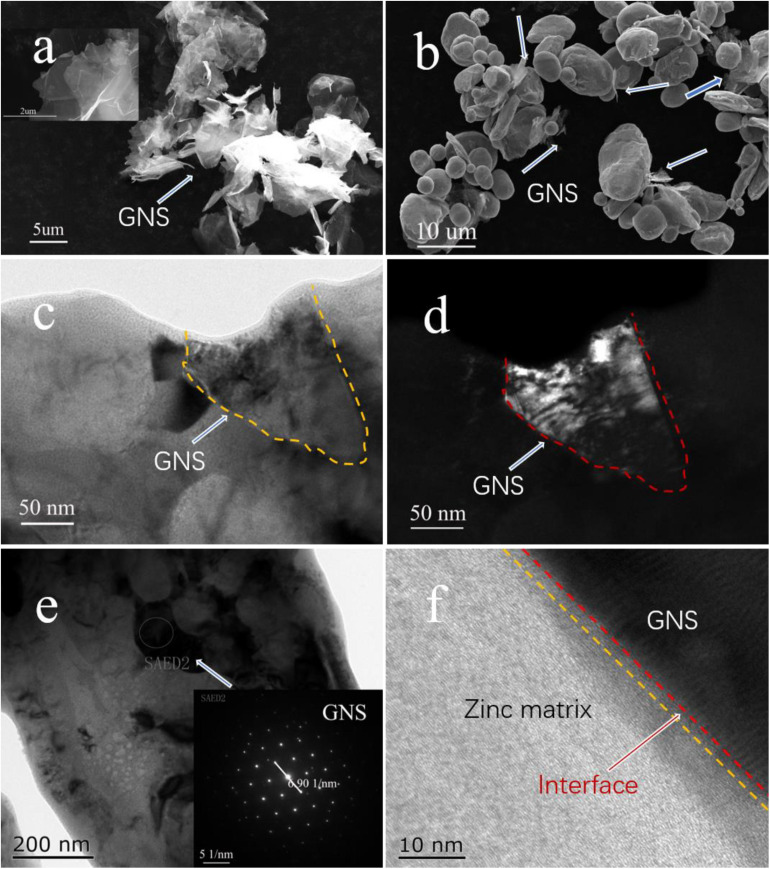
**(A)** SEM image of GNS after chemical treatment, **(B)** SEM image of ball milled powder, **(C,D)** TEM bright-field and dark-field images of GNS/Zn composite material, **(E)** TEM image of GNS displayed in composite material (inset is GNS diffraction spot), **(F)** TEM of interface relationship between Zn and GNS.

Figure 2A shows that the surface of GNS after chemical treatment changes obviously (Illustration is in the original form of GNS), which makes GNS form fold shape. This is ready for the latter work. Figure 2B shows the uniform dispersion of GNS in the matrix after ball milling, and the white arrows in the Figure indicate the position of the GNS. Figures 2C,D shows the bright-field and dark-field images of the GNS/Zn composite. The coil is the morphological feature of GNS in the matrix. Figure 2E shows the distribution of GNS in the matrix, and the selected area diffraction pattern is shown in the inset of Figure 2E. Figure 2F shows the interface relationship between GNS and the Zn matrix. From the interface diagram, it can be seen that the reinforcement GNS and the matrix Zn form a tight interface.

**FIGURE 3:**
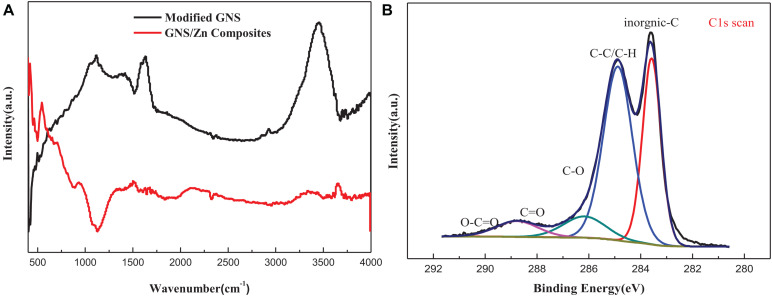
**(A)** FT-IR spectrum of modified GNS and GNS/Zn composites, **(B)** XPS spectrum of GNS/Zn composites.

Figure 3A shows the FT-IR spectra of surface-modified GNS and GNS/Zn composites. The surface-modified GNS have a broad peak near 3,473 cm^–1^ caused by the stretching vibration of alcohol and phenolic OH, and the weak peaks near 2,940 and 2,891 cm^–1^ are caused by the asymmetric and symmetrical (-CH3 group) stretching of CH deformation caused by vibration. The peak caused by the carboxyl C = O stretching vibration appears near 1,721 cm^–1^, the peak caused by the vibration deformation caused by the CC skeleton appears near 1,470 cm^–1^, and the peak near 1,025 cm^–1^ is characteristic of the S = O stretching vibration in the SDS peak (Chen et al., 2006). In the FT-IR spectrum of the composite material, the broad peak caused by the stretching vibration of OH shifted to the right from 3,473 cm^–1^ to near 3,411 cm^–1^, and the peak caused by the carboxyl C = O stretching vibration moved from 1,721 to 1,683 cm^–1^ nearby. This indicates that the modified GNS surface active groups may bond with metal ions, causing the peaks of functional groups such as OH and C = O to shift (Park et al., 2010; Wang et al., 2017). To further verify whether ionic bonds are formed. XPS analysis was performed on the composite material (Figure 3B). In the XPS peak spectrum C1s of the composite material, it can be found that several valence states of carbon have changed. At 288.2 eV, a C = O front appeared, and at 289 eV, a carbon-oxygen double bond peak appeared. Therefore, the surface-modified GNS bond with metal ions due to energy changes during the sintering process and form a tight interface, as showed in Figure 2F.

**FIGURE 4:**
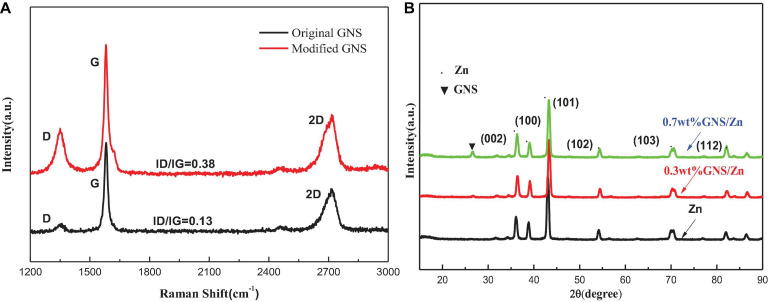
**(A)** Raman spectra of original GNS and GNSafter surface treatment, + **(B)** XRD of pure zinc and GNS/Zn composites.

Figure 4A shows the Raman spectra of the original GNS and the modified GNS. The three broad peaks in the Figure are located at the D band (defect structure) near 1,315 cm^–1^, the G band near 1,571 cm^–1^ (GNS structure) and a two-dimensional broad peak near 2,733 cm^–1^. Structural defects of the GNS can be qualitatively estimated by the intensity ratio of the D peak to the G peak (ID/IG). The lower the ratio is, the fewer the number of GNS defects (Stein et al., 2012). The high purity GNS used in this experiment can be verified by the G peak intensity. The defects of the modified GNS increase, which is exactly what the experiment need. Figure 4B shows the X-ray diffraction (XRD) pattern of the composite material obtained from the cross section of the sintered body. Compared with pure zinc, the diffraction peak of the composite material exhibits a slight change. According to the Bragg equation, a change in the lattice will cause a peak shift. Due to the addition of GNS, the internal stress during the sintering process will cause lattice distortion and lead to peak shifts. When the angle is 26.3, the diffraction peak of the GNS appears in the composite material. As the content of GNS increases, the intensity of the diffraction peak increases.

### Mechanical Properties of the Composite Materials

#### Density and Vickers Hardness of the Composite Materials

The density and Vickers hardness of pure zinc and composite materials is shown in Table 2. Adding GNS to the zinc matrix can substantially increase the hardness of the composite material. The hardness increases with increasing GNS content. When 0.7 wt% GNS is utilized, the hardness of the composite material reaches 65 HV, which is 20.3% higher than that of pure zinc (54 HV). Measured by the drainage method, the density of the composites prepared by SPS sintering is above 98%. The improvement of Vickers hardness of composites is due to rolling deformation. Because the content of reinforcement is low and evenly distributed in each position of the matrix, when hardness test is carried out, the position is randomly distributed, which is more about the strength of the matrix to bear the indenter. Therefore, the increase of hardness comes from the increase of hardness caused by rolling deformation.

**TABLE 2:** The actual density, relative density, and Vickers hardness of the materials.

Materials	Density (g/cm^3^)	Relative density (%)	Hardness before rolling (HV)	Hardness after rolling (HV)
Pure Zn	7.132 (0.012)	99.626 (0.436)	51.7 (0.3)	54.5 (0.2)
0.3 wt% GNS/Zn	7.124 (0.018)	98.737 (0.317)	53.4 (0.2)	58.7 (0.1)
0.7 wt% GNS/Zn	7.115 (0.009)	98.292 (0.151)	56.5 (0.3)	65.3 (0.2)

**Numbers in the brackets represent the standard deviation.**

#### Tensile and Compressive Properties of Pure Zinc and GNS/Zn Composites

**FIGURE 5:**
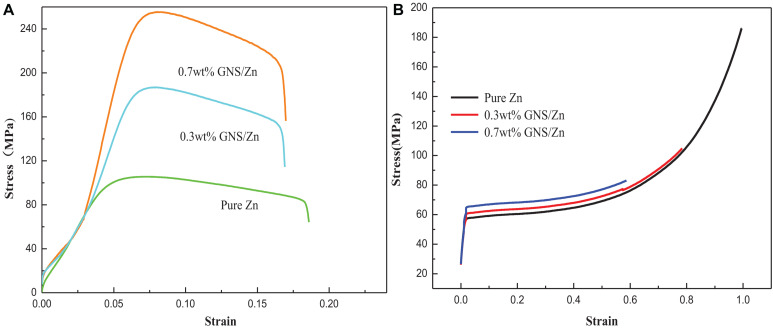
Pure zinc and GNS/Zn composites **(A)** tensile stress-strain curve, **(B)** compressive stress-strain curve.

To study the effect of the GNS content on the mechanical properties of composites, the tensile and compressive properties of composites with different GNS contents were tested. The tensile performance curve (Figure 5A) shows the effect of the GNS content on the strength and plasticity of the composites. When 0.3 wt% GNS are added, the composite material strength can reach 187 MPa, which is 67.8% higher than pure zinc (112 MPa). When 0.7 wt% GNS are added, the composite material strength can reach 254 MPa, which is 126% higher than pure zinc. It is worth noting that the tensile stress-strain diagram of the test composite material corresponds to two elastic moduli in the elastic strain stage, and its inflection point appears at approximately 50 MPa. The reason for the inflection point may be that the elastic modulus (1.1 TPa) of the reinforcement GNS used in this study is quite different from the elastic modulus of the matrix Zn (84 GPa). During the stretching process of the material, the matrix bears the external force first followed by the reinforcement. The reinforcement bears the capacity together with the matrix, so the elastic modulus of the elastic strain stage is different. Compared with pure zinc (17.13%), the elongation after fracture of the GNS/Zn composite material is reduced by less than 2%. This is because the reinforcement GNS have a larger aspect ratio. The particles will preferentially deform together with the GNS with larger specific surface areas, thereby reducing the stress concentration caused by external forces and improving the mechanical properties of the composite material. Figure 5B shows the compression curve of the composite material. It can be observed from the Figure that the compression resistance of the pure zinc and composite materials is very good. The main reason for this is that the chemically treated GNS are uniformly dispersed in the matrix and SPS sintered to form a good interface, which improves the relative density of the composite material and further improves the mechanical properties of composite materials.

**FIGURE 6:**
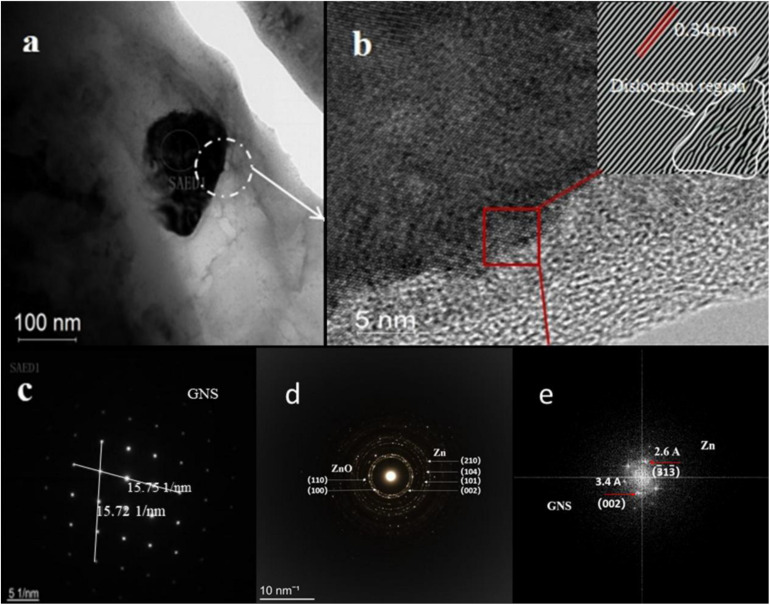
TEM micrographs of GNS/Zn composite material show **(A)** the morphological characteristics of GNS, **(B)** the typical GNS layer existing in the Zn matrix (the illustration shows the dislocation morphology view after FTT conversion), **(C,D)** is the red selected area diffraction pattern in **(A)**, **(E)** FFT pattern obtained from the area shown in **(B)**, showing inter-planar distances of graphene (3.4 Å) and Zn (2.6 Å).

Figure 6 shows a detailed TEM analysis of GNS/Zn composites. Figure 6A shows the morphological characteristics of GNS in the Zn matrix. The diffraction in the selected area is shown in Figure 6C. The GNS distributed in the matrix may improve the mechanical strength of the composite material through the dislocation pinning effect and load transfer caused by the thermal mismatch of the interface (Bhadauria et al., 2019). At the same time, the presence of GNS in the SPS sintering process will lead to partial grain refinement, thereby enhancing the strength and plasticity of the composite material. Figure 6B shows the high-resolution interface diagram of the composite material. From the perspective of the composite interface, the interface belongs to a transitional interface between tissues, which ensure the effective enhancement of the effective load transfer between the Zn matrix and the reinforcement (Bhadauria et al., 2019). Figure 6D shows the FFT image after inverse Fourier transformation. In combination with Figure 6B. It can be seen that dislocations are mainly generated at the edges and interfaces of the graphene sheet (Figure 8C2 Physical model). Figure 6E shows the FFT plot of the selected area in Figure 6B, including the GNS _002_, crystal plane and zinc matrix _331_ equal crystal plane.

#### Analysis of Tensile Fracture

**FIGURE 7:**
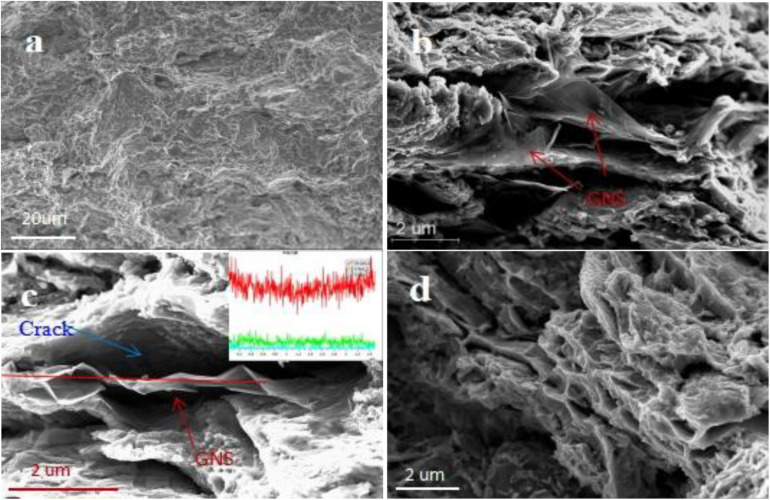
**(A)** the morphology of the tensile fracture of the GNS/Zn composite at low magnification, **(B,C)** morphological distribution of reinforcement at the fracture (the inset is a line scan of GNS), **(D)** elongated dimples morphology at the fracture of GNS/Zn composite.

Figure 7 shows the tensile fracture morphology of the composite material. Figure 7A shows that after the composite material is fractured by tension, there are a large number of tear ridges and a few quasi-cleavage steps at the fracture, which is a mixed fracture. GNS were observed in the depression of the tearing ridge, and the shape was complete. GNS mainly appeared in the way of extrusion during tensile fracture, which is the place where the crack initiated (Figures 7B,C). The load transfer of the composite material during stretching was also through the longitudinal load, which helped to improve the load transfer from the matrix to the GNS, thereby improving the tensile strength of the material (Munir et al., 2015). At the same time, a large number of stress whitening areas and elongated dimples were observed around the tear ridge (Figure 7D); that is, the material has a certain resistance to deformation, which is consistent with the tensile stress-strain curve of the material.

**FIGURE 8:**
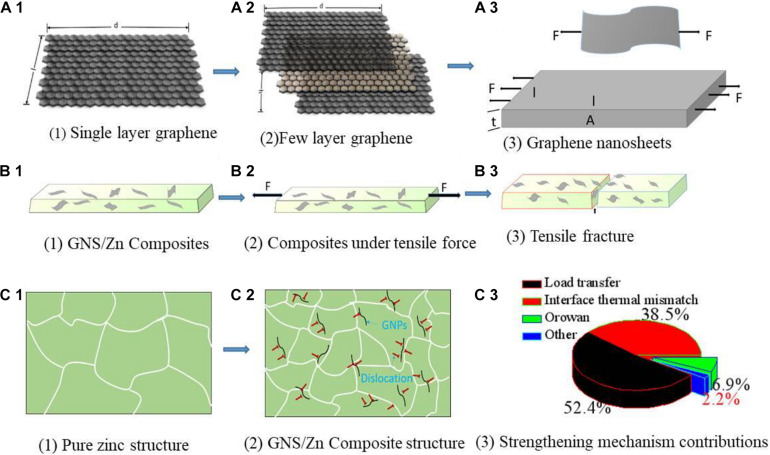
GNS/Zn composite tensile physical model **(A1–B3)**, tissue evolution process **(C1,C2)**, contribution diagram of strengthening mechanism **(C3).**

Two-dimensional GNS have a high aspect ratio, high load transfer efficiency and bridging ability (Liu et al., 2020), thereby improving the tensile strength of the composite material. When the material is subjected to an external tension, the crystal grains will preferentially deform together with the GNS with a larger specific surface area, thereby reducing the stress concentration caused by the external force. When the crack passes through the intergranular and encounters a large-sized reinforcement. The crack will accumulate near the reinforcement until the crack is blocked (Stein et al., 2012). For reinforcement distributed along the grain boundary, the microaggregation of GNS between the grains may cause stress concentrations and cracks. Therefore, it may weaken the bonding strength of the interface between the GNS and matrix.

### Strengthening Mechanism

Figure 8 shows the tensile physical model of the GNS/Zn composite and the contribution of the structure evolution process and the strengthening mechanism. The improvement in the mechanical strength of GNS/Zn composites is mainly attributed to the following aspects: (1) the load transfer of the zinc matrix to the GNS; (2) the grain refinement caused by the pinning of the GNS to the matrix; (3) the Orovan Strengthening mechanism; (4) the coefficient of thermal expansion (CTE) between Zn and GNS (Taya, 1991). Mismatches cause dislocations (Figure 8C2). The contribution of each strengthening mechanism is shown in Figure 8C3. According to the modified shear lag model, the load transfer mechanism of composite materials depends on the interface strength of the composite interface (Nardone and Prewo, 1986). The compressive yield strength of the composite material “ _σYSC_ ” can be calculated theoretically using the modified shear lag model, and the calculation formula is as follows (Nardone and Prewo, 1986):

(1) $$\sigma_{Y\,S\,C}=\sigma_{m}+\Delta \,\sigma_{L\,T}$$

(2) $$\Delta \sigma_{L\,T}=\frac{\text{s}}{4}V_{G\,N\,S}•\sigma_{\text{m}}\left(V_{G\,N\,S}=\frac{m_{G\,N\,S}}{\rho_{G\,N\,S}}•\frac{\rho_{G\,N\,S/Z\,n}}{m_{G\,N\,S/Z\,n}}\right)$$

where “_σm_” is the yield strength of pure zinc after ball milling when stretched, “_ΔσLT_” is the yield strength caused by the load transfer, “_s_” is the aspect ratio of GNS after functionalization, and “_*V_GNS*_” is the volume fraction of GNS.

The Hall-Petch equation can be used to calculate the contribution of adding GNS to the zinc matrix to grain refinement (Guo et al., 2012), and the calculation formula is as follows:

(3) $$\Delta \,\sigma_{H\,P}=K•\left({\text{d}}_{1}^{-1\,/\,2}-{\text{d}}_{2}^{-1\,/\,2}\right)$$

where “_ΔσHP_ ” is the yield strength increased by grain strengthening, “_*K*_” is the zinc Hall-Page coefficient, and “_d_” is the average grain size before and after zinc ball milling.

The CTE of GNS and Zn are “_–8×10^–6^()–1_ “ with ”_35×10^–6^()^–1^_.” The difference in the CTE value between Zn and GNS is large and the mismatch rate is high. If the yield stress of the matrix is less than the stress caused by thermal mismatch, then the composite material will form a large number of dislocations at the matrix/reinforcement interface after cooling, enhancing the effect. The following formula is used to calculate the intensity change caused by CTE mismatch (Rashad et al., 2015; Bhadauria et al., 2019):

(4) $$\Delta \,\sigma_{C\,T\,E}=4.33\,G_{\text{m}}•b\,\sqrt{\frac{\Delta \,T•\Delta \,C\,T\,E•V_{G\,N\,S}}{b•d_{G\,N\,S}}}$$

where “_*G_m*_ ” is the shear modulus of zinc, “_*b*_” is the Bernoulli vector of zinc, “_ΔT_” is the difference between the actual processing temperature and test temperature, “_ΔCTE_” is the CTE difference between Zn and GNS, and “_*d_GNS*_ ” is the film diameter of GNS.

According to Orowan’s strengthening mechanism, when a dislocation is in motion, if it encounters a hard point that cannot be crossed, then the dislocation will bypass the hard point. Therefore, when the dislocation encounters GNS, GNS will hinder the movement of the dislocation. As a result, the dislocation is forced to bypass, which will produce a back stress in the direction of the normal vector, which will further hinder the movement of the dislocation, and the dislocation packing will increase the yield stress. The Orowan equation describes the increase in the yield strength caused by the Orowan mechanism, and its calculation formula is as follows (Wang et al., 2017):

(5) $$\Delta \,\sigma_{O\,\text{r}\,o\,w\,a\,n}=\frac{0.13\,G_{\text{m}}•b}{\lambda }•I\,n\,\left(\frac{d_{G\,N\,S}}{2\,b}\right)$$

where “_Δσorowan_ ” is the change in the yield strength caused by the Orowan mechanism, “_λ_ ” is the average distance between crystal grains, and its calculation formula is:

(6) $$\lambda =d_{G\,N\,S}•\left[{\left(\frac{1}{2\,V_{G\,N\,S}}\right)}^{1\,/\,3}-1\right]$$

The effect and advantages of Aurovan's strengthening mechanism in GNS nanosheets to enhance aluminum matrix composites have been reported (Bisht et al., 2017). The calculation by the Orowan equation has a small effect on the yield strength of the matrix, so it will be ignored in this study. _Δσorowan_ is the contribution. _ΔσLT_ vs. _ΔσCTE_ depends on _*V_GNS*_ with _*d_GNS*_, _ΔσHP_ depends on the average grain size before and after ball milling, “d.” The change value of GNS is also affected by the dispersion factor of GNS in the matrix. In addition, the graphene nanoplate is used in the experiment, and the effect caused by fracture is better than that of single-layer GNS (125 GPa; Witte et al., 2007) and is substantially reduced (Figures 8B1,B2). Several strengthening mechanisms have been calculated to increase the strength of composite materials. The contribution values of _ΔσLT_, _ΔσCTE_, and _ΔσHP_ are 63, 24, and 10%, respectively (Figure 8C3). In general, two strengthening mechanisms of the GNS/Zn composites exist (Sun et al., 2019). load transfer of GNS and dislocation strengthening caused by the thermal mismatch of the interface between the reinforcement and the matrix (Figure 8). GNS’ unique two-dimensional wave structure can provide a larger interface surface area to interact with the zinc matrix, and the load transfer effect is absorbed by GNS to a greater extent and causes dislocations at the grain boundary, thus effectively strengthening the matrix.

### Biodegradability of GNS/Zn Composites

#### Electrochemical Measurements

**FIGURE 9:**
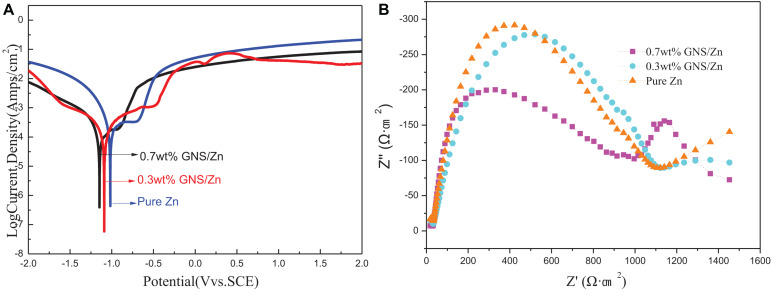
Pure zinc and GNS/Zn composites: **(A)** polarization curve, **(B)** impedance spectrum.

The short-term polarization measurement method, including the potentiodynamic polarization curve and the Nyquist plot, was used to evaluate the degradation of the experimental samples in the simulated body fluid (SBF) of the human body (Figure 9). The corrosion current density (I_*corr*_) and corrosion potential (E_*corr*_) were measured by the Tafel extrapolation method. The calculated electrochemical parameters are shown in Table 3. Compared with pure Zn, the polarization curve of the GNS/Zn composites had obvious characteristics (Figure 9A). GNS/Zn composites exhibited a certain passivation behavior during the corrosion process. The electrochemical impedance spectroscopy (EIS) of the GNS/Zn composite and pure Zn both showed a semicircular curve (Figure 9B). However, the general EIS diagram contains two semi-circular arcs corresponding to two time constants, which mainly include two capacitor circuits, a high frequency area, and a low frequency area. The capacitor ring in the high-frequency area was related to the corrosion products formed in the uniform corrosion area on the surface of the sample. The capacitance ring in the low frequency region was related to the interface charge transfer process at the sample/electrolyte interface and the electrochemical double layer effect. Semicircular diameters of GNS/Zn composites are all shorter than those of pure Zn, which indicates that their corrosion resistance is weakened. In addition, the electrochemical parameters measured by the Tafel extrapolation method also show that adding GNS to the zinc matrix will significantly impair the corrosion resistance of the composite. The main manifestations are that, as the content of GNS increases, the self-corrosion potential of the composite material decreases, the corrosion density increases, the radius of the capacitor ring in the high frequency region decreases, so the corrosion resistance of the GNS/Zn composite decreases.

**TABLE 3:** Electrochemical parameters for pure Zn and GNS/Zn composites in SBF solution.

Materials	I_*corr*_ (A/cm^2^)	E_*corr*_ (V)	Corrosion rate (mm/a)
Pure Zn	2.776 × 10^–4^ (3.548)	–1.097 (0.011)	0.069 (0.038)
0.3 wt% GNS/Zn	2.231 × 10^–4^ (2.147)*	–1.159 (0.006)*	0.213 (0.027)*
0.7 wt% GNS/Zn	1.958 × 10^–4^ (1.586)*	–1.237 (0.002)*	0.301 (0.035)*

*Numbers in the brackets represent the standard deviation*,*p < 0.05, compared with pure zinc.

#### Immersion Tests

The immersion test of the composite material was measured in SBF solution at 37°C for 36 days. During the soaking period, a pH meter was used to record the pH change of the soaking solution. After 36 days, the samples were taken out of the SBF solution. They were subsequently rinsed with demonized water and dried. Surface corrosion morphology and elements of the samples were characterized by SEM and EDS, and the composition phase of the corrosion products was identified by XRD. Then the samples were soaked in a chromic acid (CrO_3_/H_2_O) solution with a concentration of 200 g/L for 5–10 min to remove oxides and corrosion products on the surface. After ultrasonic cleaning for 20 min, they were dried and weighed. Each group was weighed three times, and the average of the results was taken. The corrosion rate was calculated according to formula (7, 8):

(7) $$v=\frac{\Delta \,m}{A\,t}$$

(8) $${\text{v}}_{s}=\frac{24\times 365}{1000}•\frac{v}{\rho }$$

“_*v*_” is the corrosion rate, “_Δm_” is the weight loss, “_ρ_ ” is the material density, “_*A*_” is the sample surface area, and “t” is the corrosion time. “_v_s_ ” is the actual measured rate.

**FIGURE 10:**
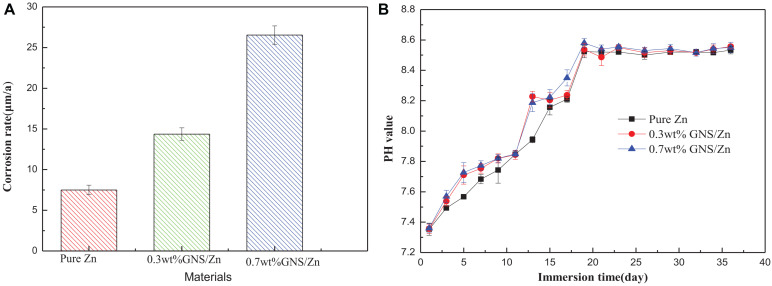
**(A)** Corrosion rate calculated by weight loss rate after 36 days of soaking. **(B)** PH change of SBF solution within 36 days of soaking.

The corrosion rate calculated from the weight loss is shown in Figure 10A. Adding GNS will accelerate the corrosion rate of composite materials in SBF solution, which is consistent with the conclusions measured by electrochemistry. The pH change during the soaking process is shown in Figure 10B. The pH changes of pure Zn and GNS/Zn composites in SBF solution are consistent. The pH value increases rapidly in the first 20 days, and the pH value of the solutions does not change much afterward. The degradation of induced pH values of GNS/Zn composites is lower than pure Zn. After 36 days soaking, the pH value of the solutions stabilizes in the region of 7.8–8.0.

The increase in pH is attributed to the alkali corrosion caused by the anion OH^–^ in the SBF solution. That is, the material will generally be oxidized to Zn^2+^ after soaking in the SBF solution (Eq. 9). The electrons generated at the anode are consumed by the corresponding cathode reaction through the reduction of oxygen to produce OH^–^ (Eq. 10). The early pH rises faster (Figure 10B). This is because Zn^2+^ and OH^–^ are released at the same time in the SBF solution, forming degradation products such as hydroxide (Eq. 11).

(9) $$\mathrm{Zn}\to {\mathrm{Zn}}^{2+}+2\,e^{-}$$

(10) $$O_{2}+2\,H_{2}\,O+4\,e^{-}\to 4\,O\,H^{-}$$

(11) $${\mathrm{Zn}}^{2+}+2\,O\,H^{-}\to \mathrm{Zn}\,{\left(\mathrm{OH}\right)}_{2}$$

However, as the immersion time is prolonged, Zn (OH)_2_ can be transformed into more stable ZnO (Eq. 12). The surface oxides generated in the neutral solution to the thermodynamic stability prediction do not form an effective anti-corrosion film. On the contrary, it is easy to further dissolve ZnO and further accelerate corrosion. When the immersion time is extended again, the pH value no longer changes significantly (Figure 10B), and the trace elements in the solution increase. In order to form a dynamic thermodynamic equilibrium, the ions in the solution will form a thermodynamically more stable ionic compound zinc phosphate tetrahedron (Formula 13).

(12) $$\mathrm{Zn}\,{\left(\mathrm{OH}\right)}_{2}\to \mathrm{ZnO}+H_{2}\,O$$

(13) $$3\,Z\,n^{2+}+2\,H\,P\,O_{4}^{2-}+2\,O\,H^{-}+H_{2}\,O\to {\mathrm{Zn}}_{3}\,{\left({\mathrm{PO}}_{4}\right)}_{2}\cdot 4\,H_{2}\,O$$

**FIGURE 11:**
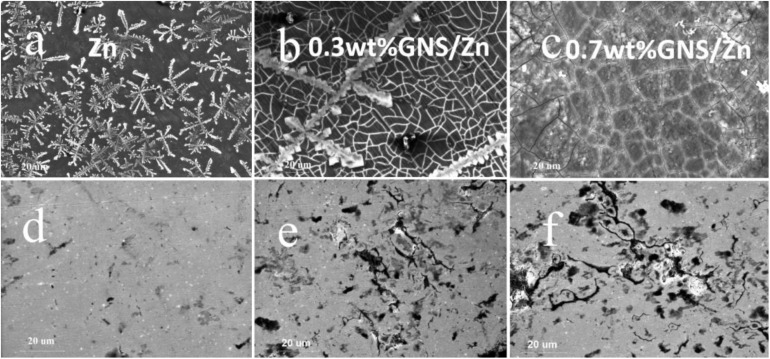
Corrosion product morphology after immersing in SBF solution for 36 days **(a)**−Pure Zn, **(b)**−0.3wt%GNS/Zn, **(c)**−0.7wt%GNS/Zn; **(d–f)** respectively correspond to **(a–c)** the topography after cleaning with chromic acid.

Figures 11A–C shows surface corrosion products distribution and morphology of pure Zn and GNS/Zn composites samples after immersing in SBF solution for 36 days. From the morphological point of view, the corrosion products formed on the pure Zn surface are less than those of the GNS/Zn composites, and the corrosion products are evenly distributed. Figures 11D–F shows the corrosion mark left on the surface of the sample after removing the corrosion products. The samples are mainly uniformly corroded in SBF solutions, and a small amount of local corrosion occurs near the GNS. The cause of local corrosion is attributed to two points: Firstly, the conductivity of GNS in SBF solution is better than that of matrix Zn. There will be a potential difference between GNS and Zn, which will cause local corrosion in this area. Secondly, because of the low sintering temperature and pressure, there was no interface reaction between GNS and the matrix Zn, the interface reaction didn’t have time to occur or it was incomplete during the sintering process, so cracks or micropores will be formed at the interface. It is easier to locally corrode at cracks or micropores during immersion.

**FIGURE 12:**
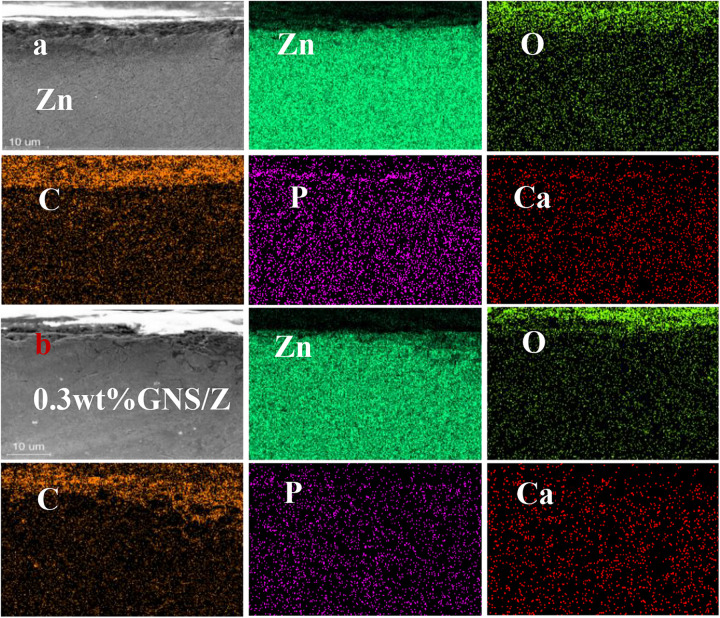
**(a)** EDS profile after immersion in pure Zn. **(b)** EDS profile of 0.3 wt% GNS/Zn composites.

**FIGURE 13:**
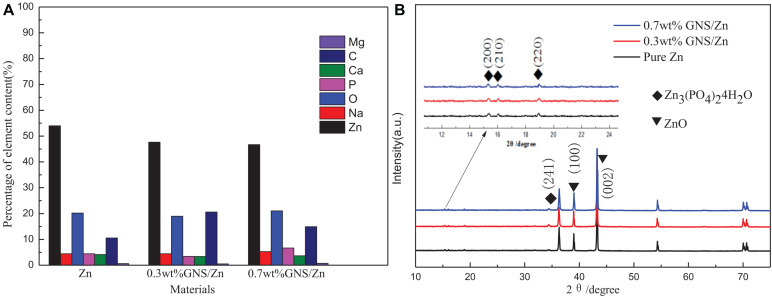
**(A)** The cross-sectional view after immersion in SBF solution corresponding to the percentage distribution of EDS element content, **(B)** XRD pattern of the corrosion products of composite materials.

Figure 12 shows the cross-sectional view of pure Zn and GNS/Zn composites after immersing in SBF solution and the corresponding EDS diagram. There is a clear boundary between the corrosion product and the matrix. Zn, O, P, Na, C, Mg, Cl, and Ca are the main elements of corrosion products, and the distribution of the elements is uniform, which proves that corrosion is more inclined to uniform corrosion. After adding GNS, the EDS diagram of the composites does not change much, but from the cross-sectional view, the depth of the corrosion layer of the composite material is deeper than that of pure zinc. Figure 13A shows the mass percentage of the corresponding elements in the EDS diagram. As the content of GNS increases, the proportion of zinc element decreases, and the proportion of the corresponding corrosion products element content increases. In order to further study the chemical composition of the corrosion product, XRD was used to detect the corrosion products (Figure 13B). It could be seen that ZnO and Zn_3_ (PO4)_2_⋅4H_2_O were the main crystalline products. The XRD did not detect the presence of the CaCO_3_ crystalline product, and the content of Ca in the EDS diagram was not low, indicating that Ca mainly existed in the form of ions and could be absorbed by the SBF solution. The results of EDS and XRD provided an effective basis for the change of the surface state of the material in the SBF solution.

In summary, the biodegradability of GNS/Zn composites was evaluated by electrochemical measurement and immersion test. The results show that adding GNS to the matrix will increase the degradation rate of the composites. The rate of degradation can be achieved by adjusting the mass percentage of GNS. The degradation rate measured by the Tafel extrapolation method is consistent with the actual degradation rate value of the immersion test. Its degradation rate is in the range of 69–301μm/a, which is an ideal degradation rate as an orthopedic implant material (Erinc et al., 2009). In addition, the GNS/Zn composites corrode uniformly in the SBF solution as a whole, but corrode near the GNS locally. By analyzing the EDS of the sample section after immersion and the XRD of the corrosion products, it has been found that the corrosion products are mainly Zn_3_(PO4)_2_⋅4H_2_O crystals.

## Conclusion

The GNS/Zn composites were successfully prepared using SPS technology. Compared with pure zinc, the mechanical properties of GNS/Zn composites are significantly improved. When 0.7 wt% GNS are added, the ultimate tensile strength of the composite material is 254 MPa, the hardness is 65 HV, which is 126 and 22.3% higher than pure Zn (112 MPa, 45 HV), respectively, and the elongation at the breaking point is above 15%. The load transfer effect of GNS and dislocation strengthening caused by interface thermal mismatch are the main strengthening mechanisms of GNS/Zn composites. GNS composites are mainly uniformly corroded in SBF solution, and a small amount of local corrosion occurs near GNS. In addition, adding GNS to the matrix can accelerate the degradation rate of the composite material, but the degradation rate can be controlled by the content of GNS, and the degradation rate is between 69 and 301 μm/a.

## Data Availability Statement

The raw data supporting the conclusions of this article will be made available by the authors.

## Author Contributions

QD, FZ, SP, ZZ, YL, and MF: writing-original draft and writing-review and editing. All authors contributed to the article and approved the submitted version.

## Conflict of Interest

The authors declare that the research was conducted in the absence of any commercial or financial relationships that could be construed as a potential conflict of interest.
